# *Citrus aurantium* L. and synephrine improve brown adipose tissue function in adolescent mice programmed by early postnatal overfeeding

**DOI:** 10.3389/fnut.2023.1278121

**Published:** 2024-01-11

**Authors:** Andressa Cardoso Guimarães, Egberto Gaspar de Moura, Stephanie Giannini Silva, Bruna Pereira Lopes, Iala Milene Bertasso, Carla Bruna Pietrobon, Fernanda Torres Quitete, Tayanne de Oliveira Malafaia, Érica Patrícia Garcia Souza, Patrícia Cristina Lisboa, Elaine de Oliveira

**Affiliations:** ^1^Laboratory of Physiology of Nutrition and Development, Department of Physiological Sciences, Roberto Alcantara Gomes Biology Institute, State University of Rio de Janeiro, Rio de Janeiro, Brazil; ^2^Laboratory of Endocrine Physiology, Department of Physiological Sciences, Roberto Alcantara Gomes Biology Institute, State University of Rio de Janeiro, Rio de Janeiro, Brazil; ^3^Laboratory for Studies of Interactions Between Nutrition and Genetics, Department of Basic and Experimental Nutrition, State University of Rio de Janeiro, Rio de Janeiro, Brazil

**Keywords:** obesity, adolescence, metabolic programming, early overfeeding, *Citrus aurantium*, synephrine

## Abstract

**Introduction and aims:**

Obesity is a multifactorial condition with high health risk, associated with important chronic disorders such as diabetes, dyslipidemia, and cardiovascular dysfunction. *Citrus aurantium* L. (*C. aurantium*) is a medicinal plant, and its active component, synephrine, a β-3 adrenergic agonist, can be used for weight loss. We investigated the effects of *C. aurantium* and synephrine in obese adolescent mice programmed by early postnatal overfeeding.

**Methods:**

Three days after birth, male Swiss mice were divided into a small litter (SL) group (3 pups) and a normal litter (NL) group (9 pups). At 30 days old, SL and NL mice were treated with *C. aurantium* standardized to 6% synephrine, *C. aurantium* with 30% synephrine, isolated synephrine, or vehicle for 19 days.

**Results:**

The SL group had a higher body weight than the NL group. Heart rate and blood pressure were not elevated. The SL group had hyperleptinemia and central obesity that were normalized by *C. aurantium* and synephrine. In brown adipose tissue, the SL group showed a higher lipid droplet sectional area, less nuclei, a reduction in thermogenesis markers related to thermogenesis (UCP-1, PRDM16, PGC-1α and PPARg), and mitochondrial disfunction. *C. aurantium* and synephrine treatment normalized these parameters.

**Conclusion:**

Our data indicates that the treatment with *C. aurantium* and synephrine could be a promising alternative for the control of some obesity dysfunction, such as improvement of brown adipose tissue dysfunction and leptinemia.

## 1 Introduction

Obesity is considered a chronic and multifactorial disease characterized by the excessive accumulation of body fat, which result from a complex interaction of genetic, metabolic, social, behavioral, and psychological factors (1). Currently, obesity is a worldwide public health problem as it is associated with an elevated susceptibility of chronic diseases, such as diabetes, dyslipidemia, cardiovascular impairment, and cancer (2, 3). Obesity pathophysiology includes insulin resistance, chronic inflammation, oxidative stress, and dysfunctional angiogenesis (3, 4). Childhood overweight and obesity have tripled since the 1970s, and rates of severe obesity more than quintupled in the same period (5), demonstrating an increase in obesity in childhood and adolescence in recent years. Concomitantly, it was observed that changes in dietary patterns, decreased physical activity (6), socioeconomic influences, and physiological stress (5) create an obesogenic environment.

Cohort studies show that overweight in childhood is a predictor of obesity in adulthood, with high correlations with body mass index (7, 8). Therefore, children and adolescents with overweight/obesity are more likely to become obese when adults, reducing their quality of life and life expectancy (8, 9).

Pregnancy, lactation, and adolescence are considered susceptible life critical windows to metabolic programming. During these stages, alterations in nutritional, hormonal and the environment can promote morphological and functional adjustments that may increase the predisposition to development of diseases throughout life (10). This concept is called Developmental Origins of Health and Disease (DOHaD) (11).

Early postnatal overfeeding model, induced by litter size reduction, causes changes in brown adipose tissue (BAT), reducing the expression of transcriptional regulators, such as PPARγ (peroxisome proliferator-activated receptor gamma) and C/EBP (CCAAT/enhancer binding proteins), and reducing lipase, sympathetic β3-adrenergic receptor, and UCP-1 (uncoupling protein 1) gene expression on PND60 (12, 13). Dysfunctional BAT shows lower thermogenic activity characterized by lower UCP1 expression and increased lipid droplet accumulation (13, 14). In addition, the litter size reduction model in rodents has several short- and long-term pathophysiological outcomes, including increased adiposity, high concentrations of leptin, insulin, and glucocorticoids, and leptin and insulin resistance. These factors collectively contribute to cardiovascular dysfunction and type 2 diabetes (12, 15, 16).

Adolescence represents a period of accelerated growth, characterized by increased nutritional requirements, physiological increases in adipose tissue, and enhanced consumption of fast food (5, 17). Moreover, the increase in brain plasticity during this critical window may predispose an adolescent responsive to interventions, which could help to compensate for or attenuate earlier developmental insults (18). For all these reasons, adolescence is critical to initiate and aggravate several disorders, such as binge eating, leading to obesity.

Although obesity in childhood and adolescence has been increasing significantly in recent years, studies have shown that current strategies for obesity management in this age group are ineffective, showing the importance of searching for new treatment alternatives (19). Since obesity is characterized by abdominal fat accumulation, recent studies search for novel therapeutic approaches using natural compounds, including herbal medicines and medicinal plants. These substances have the potential to reduce adiposity and/or increase thermogenesis through BAT modulation. Such modulation has a direct and beneficial effect on glucose and lipid metabolism, regardless of the effect on body weight (20, 21). In addition, natural compounds generally have fewer adverse effects (20).

*Citrus aurantium* L. (*C. aurantium*) comes from small fruit trees approximately five meters tall and with scented white flowers (22) belonging to the Rutaceae family and popularly known for bitter orange, sour orange, and Seville orange (23). Due to their medicinal properties, products derived from *C. aurantium* are commonly used as medicine and in dietary supplements (24). Its peel and/or immature fruits are used in health applications, such as digestive (25), anxiolytic, insomnia relief (26), antioxidant, anti-inflammatory (27) and adjuvant applications in obesity (24). The plant’s unripe dry fruit contains approximately 10% flavonoids (naringin, hesperidin, neohesperidin, limonene, tangeretin, and nobiletin, carotenoids) and phenylethylamines (methyltyramine, tyramine, octopamine, and, mainly, synephrine) (22, 24).

In 2004, the Food and Drug Administration (FDA) banned the use of ephedra, derived from *Ephedra sinica*, in dietary supplements in the United States due to its clinical association with heart and central nervous system problems (28). Synephrine is a chiral amine that is present in nature in the form (R)–(-) -p-synephrine (or l-synephrine) (28, 29) and a compound chemically similar to ephedrine, presenting with a similar structural composition and differing only by a hydroxyl ring in the para position of the benzene ring (30) and a methyl group on the side chain (CH3) present in ephedrine (28). As ephedrine, synephrine is a β3 adrenergic agonist receptor with thermogenic and lipolytic actions (31). This structural variation alters the pharmacokinetics, resulting in fewer adverse effects on heart rate and blood pressure than ephedrine. Thus, the use of synephrine as a substitute for ephedrine has become more frequent (32). p-Synephrine is present in greater quantities in bitter orange fruit peel and is the main active component of *C. aurantium* (23).

*Citrus aurantium* and synephrine are known for their therapeutic potential in thermogenesis stimulation. However, studies that indicate *C. aurantium* and synephrine as inducers of weight loss and thermogenic action in adipose tissue are still scarce (33). Moreover, the most of them are carried out in combination with other medicines and plants or to assess its toxicity (28, 34, 35).

Considering all the aforementioned factors, we hypothesized that adolescence can be considered an important window of opportunity for the implementation of anti-obesity therapeutic strategies (18, 36). Therefore, new treatment alternatives that are more efficient for obesity management should be studied. Here, we investigated the effectiveness of *C. aurantium* and/or of synephrine on brown adipose tissue dysfunction and metabolic disorders in adolescent obese mice programmed through early postnatal overfeeding. This research aims to assess their potential role as a method for metabolic “deprogramming” and improve our understanding of the mechanisms involved in the anti-obesity action of this medicinal plant.

## 2 Materials and methods

The experimental protocol was approved by the Animal Care and Use Committee of the Biology Institute of the State University of Rio de Janeiro (CEUA/009/2018).

The sample size was calculated based on previous experimental findings that have robustly demonstrated statistically significant increases in biometric parameters, such as body mass and adiposity, relative to the control group (37). In adherence to the principles of the 3 Rs model (reduction, refinement, replacement), we also sought to minimize the utilization of animals while still preserving statistical significance. Additionally, we accounted for a potential 10% attrition rate to accommodate any unanticipated contingencies.

### 2.1 Experimental model of litter size reduction

The Swiss mice used in the experiment were housed under control temperature (25°C ± 1°C), light (12 h light/dark cycle), and with free access to water and food. Three-month-old male and female nulliparous mice were mated in a 2:1 ratio for 7 days. The pregnant females (*n* = 30) were kept individually in cages until delivery. After birth, the litters were adjusted to 9 pups per mother. To induce early overfeeding (small litter group – SL), on postnatal Day 3 (PND3), the litter size was reduced to 3 pups per mother (18 mothers). The control group (normal litter group–NL) was maintained with 9 pups per mother (12 mothers) until weaning (PND21), when reduced to 6 animals per group. On PND21, the SL and NL groups were subdivided into 4 groups (10–12 animals per group):

(1) The SL group was subdivided into a small litter group treated with vehicle (distilled water) (SL), a small litter group treated with *C. aurantium* standardized to 6% synephrine (SL-CA6%), a small litter group treated with *C. aurantium* standardized to 30% synephrine (SL-CA30%), and a small litter group treated with isolated synephrine (SL-Syn).
(2) The NL group was subdivided into a normal litter group treated with vehicle (distilled water) (NL), a normal litter group treated with *C. aurantium* standardized to 6% synephrine (NL-CA6%), a normal litter group treated with *C. aurantium* standardized to 30% synephrine (NL-CA30%), and a normal litter group treated with isolated synephrine (NL-Syn).

Only male mice were used in the whole experiment. All groups were treated with their respective doses administered by gavage, during PND30 to PND49, that correspond to the period of adolescence in mice (38) (Supplementary Table 1). All groups received the same volume through gavage (350 ul). The doses of *C. aurantium* and synephrine were based on the descriptions by Deshmukh et al. (39).

The extracts of *C. aurantium* standardized at 6 and 30% synephrine were obtained from the Manipulation Pharmacy Caminhoá from China (lot numbers 206171201 and HK18050201, respectively). Isolated synephrine was obtained from Sigma Aldrich (lot BCBW4296 and code 75256). All 3 treated groups received the same amount of synephrine (1.5 mg/kg of weight/day).

**FIGURE 1:**
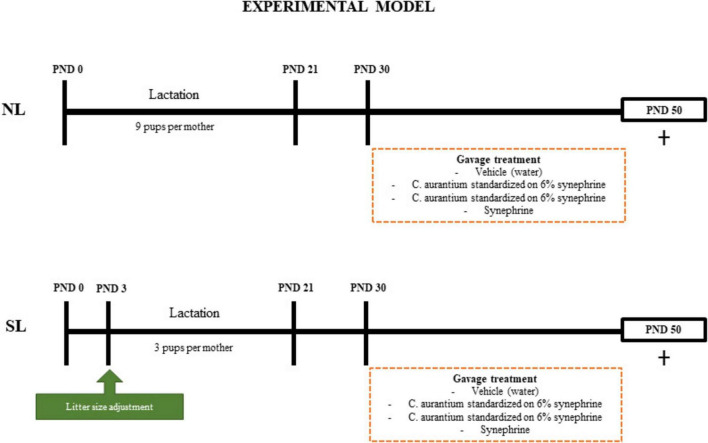
Experimental model. Postnatal day (PND). Males (♂). Mice raised in normal litter (NL); Mice raised in normal litters treated with *C. aurantium* standardized on 6% synephrine (NL-CA6%); Mice raised in normal litters treated with *C. aurantium* standardized on 30% synephrine (NL-CA30%); Mice raised in normal litters treated with synephrine (NL-Syn); Mice raised in small litter (SL); Mice raised in small litters treated with *C. aurantium* standardized on 6% synephrine (SL-CA6%); Mice raised in small litters treated with *C. aurantium* standardized on 30% synephrine (SL-CA30%); Mice raised in small litters treated with synephrine (SL-Syn).

A summary of the experimental model is shown in Figure 1.

### 2.2 Body weight measurement

During the lactation period (21 days of life), the animals were weighed daily. After weaning, the animals were weighed every 3 days on a mini digital weight scale (Professional digital weight scale MOD 1200).

### 2.3 Body composition analysis

Body composition was analyzed using whole-body nuclear magnetic resonance (NMR) imaging (Minispec LF90 TD-NMR, Bruker, Rheinstetten, Germany) in the pretreatment and posttreatment periods to evaluate total fat mass. The non-anesthetized animals were placed in a transparent plastic cylinder and kept immobile due to the insertion of a very tight plunger in the cylinder. Soon after, the cylinder with the animal was inserted into the NMR chamber, remaining during the examination for approximately 2 min. The data were expressed in grams (g) of adipose mass.

### 2.4 Analysis of cardiovascular parameters

Systolic blood pressure, diastolic blood pressure, and heart rate were assessed using a non-invasive method (Tail-cuff plethysmograph- LE5001 Panlab, Barcelona, Spain). The animals were acclimatized for 2 days, and then, the animals were submitted to the procedure again. The measurements were recorded and averaged.

### 2.5 Oral glucose tolerance test (OGTT)

The oral glucose tolerance test was performed 2–3 days before euthanasia (47–48 days of life) using a glucometer (ONETOUCH ULTRA^®^, Johnson & Johnson, São Paulo, Brazil). After 12 h of fasting, blood samples were collected to assess baseline glycaemia (time 0). Then, glucose (50%) was administered in sterile saline (0.9% NaCl) by gavage. Glucose was measured at 15, 30, 60, and 120 min after glucose administration.

### 2.6 Euthanasia and tissue collection

At 50 days of age, after a 6-h fasting period (07:00–12:00 h), the animals were anesthetized with avertin (2,2,2–Tribromoethanol, 2-methyl-2-butanol – 0.02 ml/g body weight) and euthanized by cardiac puncture. Blood samples were collected in a vacuum tube with heparin and centrifuged (1,000 × *g*, 4°C, 20 min) to obtain plasma, which was stored at −20°C until analysis. The BAT was dissected, weighed, and prepared for morphological and molecular analysis (real-time PCR). The adrenal glands were frozen to assess the adrenal catecholamine content.

### 2.7 Plasma analysis

Leptin, corticosterone, and thyroid hormones (total T3 and free T4) were analyzed from the animals’ plasma using an ELISA kit (EMD Millipore Corporation). The reading took place on the PerkinElmer EnVision Wallac-2104 Multilabel Reader, following the manufacturer’s instructions.

### 2.8 Adrenal catecholamine measurement

The right adrenal tissue stored in acetic acid was used for analysis. The adrenals were homogenized in 10% acetic acid and centrifuged (4°C, 1120 × *g* for 5 min). The subsequent steps were performed as previously described (13).

### 2.9 Morphological evaluation of BAT

On the day of euthanasia, BAT fragments were washed in buffered saline solution (PBS) and immersed in 4% paraformaldehyde and 4% paraformaldehyde + 10% sucrose for 30 min each. After that, the BAT fragments were submerged in 20% sucrose phosphate buffer in the refrigerator. From each tissue, non-serial sections 5 μm thick were obtained (microtome Microtec-CUT 4050, SC, USA). The sections were placed on glass slides for staining with hematoxylin/eosin.

Digital images were acquired randomly (TIFF format) using an Olympus DP71 camera coupled to an Olympus BX40 light microscope (Olympus, Japan). Ten photomicrographs per animal were used. BAT digital images were analyzed, and their areas were calculated. All photomicrographs were measured with Image-Pro Plus 5.0 software (Media Cybernetics, USA).

### 2.10 RT–qPCR

Total RNA was extracted from **BAT** samples using the RNeasy Lipid Tissue kit (Qiagen, Germantown, Maryland) following the protocol described by the manufacturer. The integrity of the RNA was evaluated by electrophoresis on a 2% agarose gel (containing 1.5 μl of Red Gel), allowing the visualization of the 28 S and 18 S subunits of the RNA in ultraviolet light (UV). The RNA quantification was evaluated with a spectrophotometer (NanoVue Plus, GE Healthcare^©^ New Jersey, USA), and the absorbance ratios of 260/280 nm were considered satisfactory between 1.8 and 2.0.

cDNA was synthesized using a reverse transcription kit (Applied Biosystems Thermo Fisher Scientific, Massachusetts, USA), and the samples were incubated in a thermocycler (Applied Biosystems Veriti 96 Well Thermal Cycler). The primers were purchased from TaqMan Thermo Fisher Scientific (Supplementary Table 2). In each reaction plate, the negative control without sample (C-), the negative control without enzyme (RT-), and the standard curve of serial dilution corresponding to the gene of interest were added.

The results were expressed in relation to the expression values of their control groups, which were 1 and normalized to the standard curve. Subsequently, we used these values for statistical analysis. The efficiencies of each test were calculated from a serial dilution curve present on each plate, using only plates whose efficiencies were between 85 and 110.

### 2.11 BAT mitochondrial function

Brown adipose tissue respiration was determined as previously described (40, 41) with minor modifications. BAT was prepared for measurements of respiratory flux rates by mechanic dissection with sharp forceps in relaxing buffer (BIOPS; in mM: CaK2EGTA 2.77, K2EGTA 7.23, MgCl2 6.56, dithiothreitol 0.5, K-MES 50, imidazole 20, taurine 20, Na2ATP 5.77, phosphocreatine 15, pH 7.1 adjusted at 25°C) on ice. After that, the interscapular brown adipose tissues were washed in ice-cold respiration medium (MIR05–in mM: EGTA 0.5, MgCl2 3.0, K-MES 60, taurine 20, K2HPO4 10, HEPES 20, Sucrose 110 and BSA 1 g/L, pH 7.1 adjusted at 25°C) for 10 min under agitation.

The respiratory rates of BAT were determined with the Oroboros 2k-Oxygraph (Oroboros Instruments, Innsbruck, Austria) in 2 ml of MIR05 at 37°C with continuous stirring. Before adding the tissue into the chamber, wet weight measurements were taken, and a sample of 5–7 mg was used per chamber. All measurements were taken at oxygen concentrations above 400 nmol ml-1 in the chamber. DatLab software (Oroboros Instruments, Innsbruck, Austria) was used for data acquisition and analysis. Oxygen consumption rates are expressed as pmol of O2⋅ s-1⋅mg wet weight-1. Digitonin is used to permeabilize the cell membranes while leaving the mitochondrial membranes intact because of its specificity for solubilizing cholesterol, which exists in much higher concentrations in the plasma membrane. The study was carried out with two groups of independent substrates in each chamber: (chamber A, in mM) glutamate 10, pyruvate 5, malate 2, ADP 1 and succinate 10, for the analysis of carbohydrate-related oxidation (with electron entry through complexes I and II of the respiratory chain) and (chamber B, in mM) palmitoyl-carnitine 0.02, malate 2 and ADP 5, for the analysis of oxidation related to fatty acids.

Respiratory parameters were defined as: state 1–basal respiratory rate, without addition of substrates; state 2–before addition of adenosine diphosphate (ADP); state 3 (Complex I)–maximum respiratory rate stimulated by ADP (5 mM); state 3 maximum (Complex I **+** II)–after addition of succinate and state 4, respiration rates after the addition of the Adenosine triphosphate (**ATP**) synthase inhibitor oligomycin (1 μg/ml) to identify proton leaks from the intermembrane space into the mitochondrial matrix. From the respiratory fluxes obtained during the substrate titration protocol, the respiratory control ratio (**RCR**) was calculated for State 3/State 4, which was used as a general measure of mitochondrial function. The addition of cytochrome c (10 μM) allowed for the evaluation of the integrity of the mitochondrial membrane because an increase in respiration with the addition of cytochrome c indicates a defect in the outer mitochondrial membrane (42).

### 2.12 Statistical analysis

Statistical analyses were performed using GraphPad Prism software (version 6.00, Rio de Janeiro, Rio de Janeiro, Brazil) and expressed as the mean ± standard error of the mean. Statistical analysis of body weight evolution during lactation period was performed using One-Way ANOVA followed by Fisher’s LSD test for multiple comparisons. Statistical analysis of pretreatment NL and SL groups of body composition by NMR at 30 days of life was performed using Student’s unpaired *t*-test analysis. Statistical analysis of high resolution respirometry was performed using One-Way ANOVA followed by Tukey’s test for multiple comparisons. The other analyses included bivariate analysis of variance (two-way ANOVA) followed by Tukey’s test for multiple comparisons or Mann-Whitney U test to non-parametric parameters. Differences were considered significant when *p* < 0.05.

## 3 Results

### 3.1 Body weight during lactation

**FIGURE 2:**
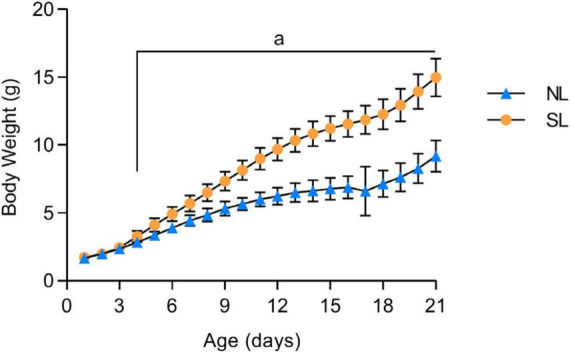
Evolution of body weight during the lactation period (21 days) of mice raised in normal (NL) and small (SL) litters. Results analyzed by bivariate analysis of variance (one-way ANOVA) followed by *post-hoc* Fisher’s LSD test and expressed as mean ± SD.; *n* = 50–70 per group; *p* < 0.05; *a* = vs. NL.

The body weights of normal litters (NL) and small litters (SL) during lactation (PND21) are shown in Figure 2. The small litter group (SL) had a higher body weight than the normal litter group (NL) on PND4 until weaning (PND21, SL: 3.3 ± 0.06 vs. NL: 2.8 ± 0.03; *p* = 0.0001).

### 3.2 Body weight and fat mass

**FIGURE 3:**
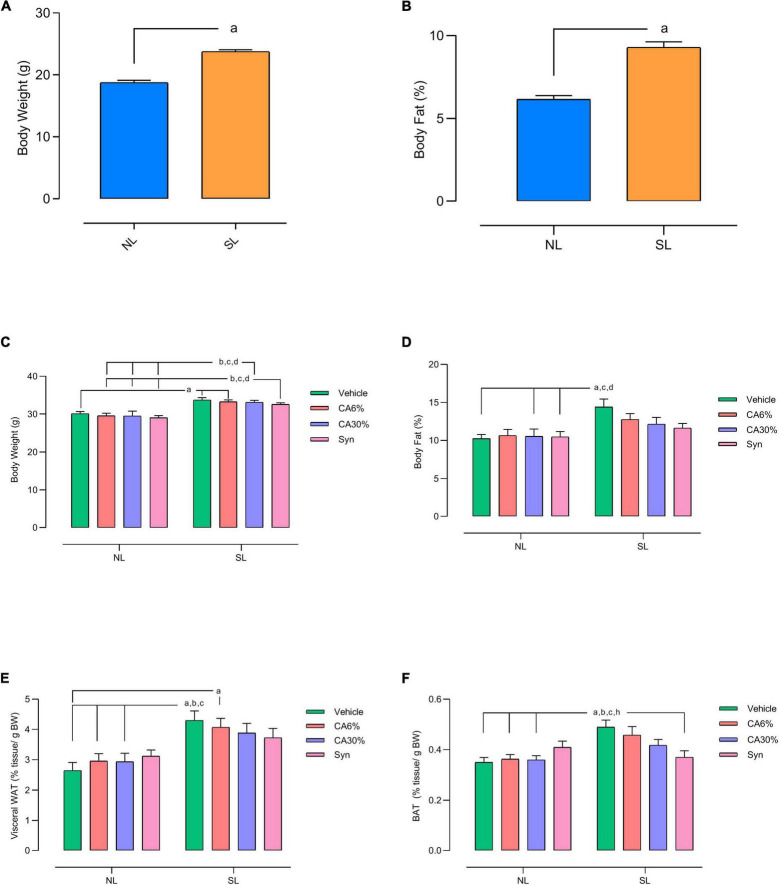
Effect of treatments with *C. aurantium* and synephrine on body weight **(A,C)**, body composition by Nuclear Magnetic Resonance **(B,D)** and tissue weight of visceral WAT **(E)** and BAT **(F)** of mice raised in normal and small litters in the pre-treatment **(A,B)** and post-treatment **(C–F)** period. Results analyzed by Student’s unpaired *t*-test and expressed as mean ± SEM; *n* = 32–42 per group; *p* < 0.05; *a* = vs. NL **(A,B)**. Results analyzed by bivariate analysis of variance (two-way ANOVA) followed by Tukey’s multiple comparisons test and expressed as mean ± SEM; *n* = 7–11 per group; *p* < 0.05; *a* = vs. NL, *b* = vs. NL-CA6%, *c* = vs. NL-CA30%, *d* = vs. NL-Syn **(C,D)**. Results analyzed by bivariate analysis of variance (two-way ANOVA) followed by Tukey’s multiple comparisons test and expressed as mean ± SEM; *n* = 10–12 per group; *p* < 0.05; *a* = vs. NL, *b* = vs. NL- CA6%, *c* = vs. NL- CA30% and *h* = vs. SL-Syn **(E,F)**.

The SL group had a higher body weight (NL: 18.8 ± 0.36 vs. SL: 23.8 ± 0.29; *p* = 0.0001) (Figure 3A) and greater fat mass (NL: 2.0 ± 0.06 vs. SL: 3.1 ± 0.09; *p* = 0.0001) (Figure 3B) than the NL group at PND 29 in the pretreatment period with *C. aurantium* and synephrine. In the posttreatment period (PND49), SL groups have higher body weights than all NL groups (*p* ≤ 0.01) (Figure 3C). The SL group also had higher fat mass than the normal litter groups (NL, NL-CA30%, NL-Syn) (*p* ≤ 0.05) (Figure 3D). However, the SL groups treated with 6 or 30% *C. aurantium* and synephrine did not show significant differences in fat mass with the NL groups (Figure 3D).

The SL and SL-CA6% groups had a higher visceral WAT weight than the NL groups (*p* ≤ 0.01) (Figure 3E). BAT weight was increased in the SL group compared to the NL groups (*p* ≤ 0.05). However, treatment with synephrine (SL-Syn) reduced the BAT weight compared to the SL group (−26% vs. SL; *p* ≤ 0.05) (Figure 3F). The SL-CA30% and SL-Syn groups showed no difference in visceral WAT weight (Figure 3E) and BAT weight (Figure 3F) from the NL groups.

### 3.3 Accumulated body weight gain

**FIGURE 4:**
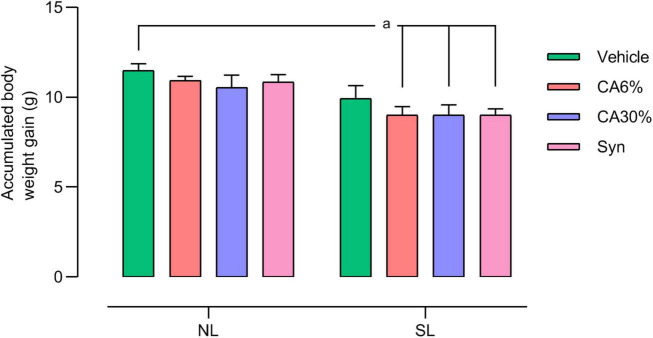
Accumulated weight gain of mice raised in normal and small litters submitted to treatment with *C. aurantium* and synephrine. Results analyzed by bivariate analysis of variance (two-way ANOVA) followed by Tukey’s multiple comparisons test and expressed as mean ± SEM; *n* = 10–12 per group; *p* < 0.05; *a* = vs. NL.

The accumulated body weight from PND30 to PND49 is depicted in Figure 4. The SL groups treated with *C. aurantium* (SL-CA6 and SL-CA30%) and synephrine (SL-Syn) had less body weight gain than the NL group (*p* ≤ 0.05).

### 3.4 Cardiovascular parameters

There was no significant difference in heart rate (Supplementary Figure 1A), systolic blood pressure (Supplementary Figure 1B), and diastolic blood pressure (Supplementary Figure 1C) in the normal and small litter groups treated with *C. aurantium* and synephrine or vehicle.

### 3.5 Oral glucose tolerance test

There was no significant difference in the OGTT (Supplementary Figure 2A) or the area under the curve (AUC) of the OGTT (Supplementary Figure 2B) in the normal and small litter groups treated with *C. aurantium* and synephrine or vehicle.

### 3.6 Plasma hormonal analysis

**FIGURE 5:**
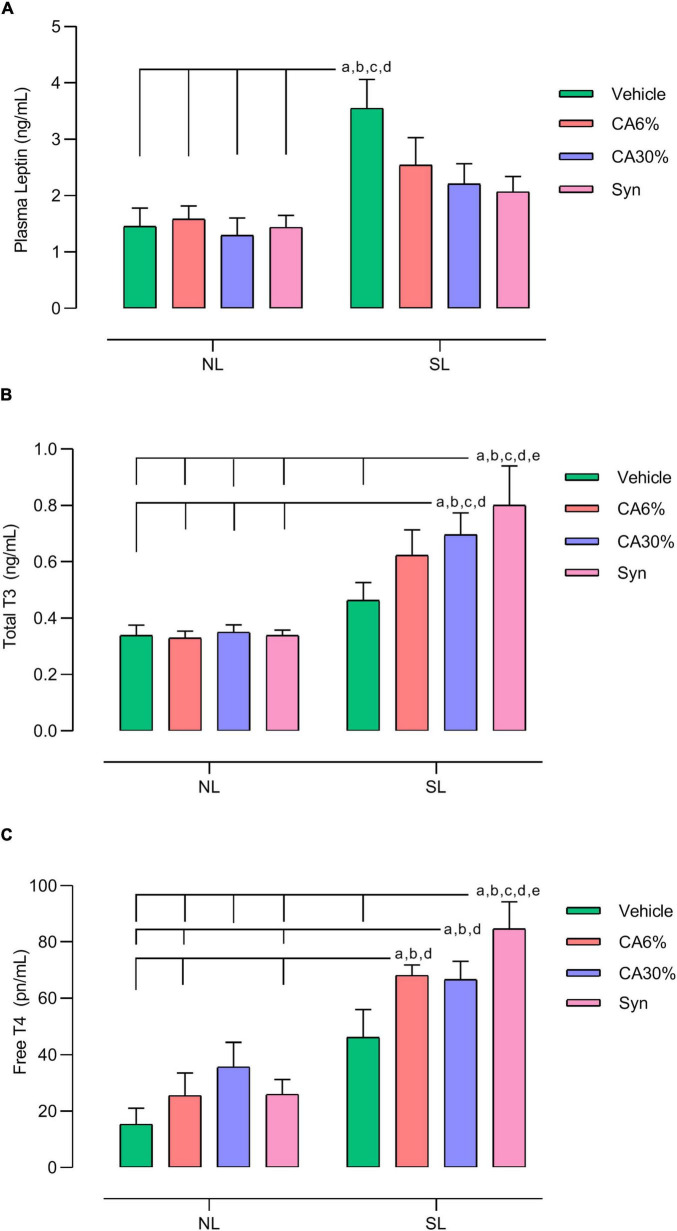
Effect of treatment with *C. aurantium* and synephrine in hormonal dosages. Plasma leptin **(A)**, Total T3 **(B)** and Free T4 **(C)**. Results analyzed by bivariate analysis of variance (two-way ANOVA) followed by Tukey’s multiple comparisons test and expressed as mean ± SEM; *n* = 8–10 per group; *p* < 0.05; *a* = vs. NL, *b* = vs. NL-CA6%, *c* = vs. NL-CA30%, *d* = vs. NL-Syn; *e* = vs. SL.

The SL group had a higher plasma concentration of leptin than all NL groups (NL, NL-CA6%, NL-CA30%, NL-Syn) (*p* ≤ 0.001). The plasma concentration of leptin in the SL groups treated with *C. aurantium* and synephrine was not different from that in the NL groups or SL vehicle group (Figure 5A). The SL-CA30% and SL-Syn groups had higher total plasma T3 concentrations than the NL groups (*p* ≤ 0.05). Only the SL-Syn group had increased total plasma T3 concentration compared to the SL group (*p* ≤ 0.05) (Figure 5B). The SL-CA6%, SL-CA30%, and SL-Syn groups had higher plasma free T4 concentrations than the NL groups (*p* ≤ 0.01) (Figure 5C). Only the SL-Syn group had an increased plasma free T4 concentration compared to the SL group (*p* ≤ 0.05) (Figure 5C).

### 3.7 Tissue catecholamine and plasma corticosterone

**FIGURE 6:**
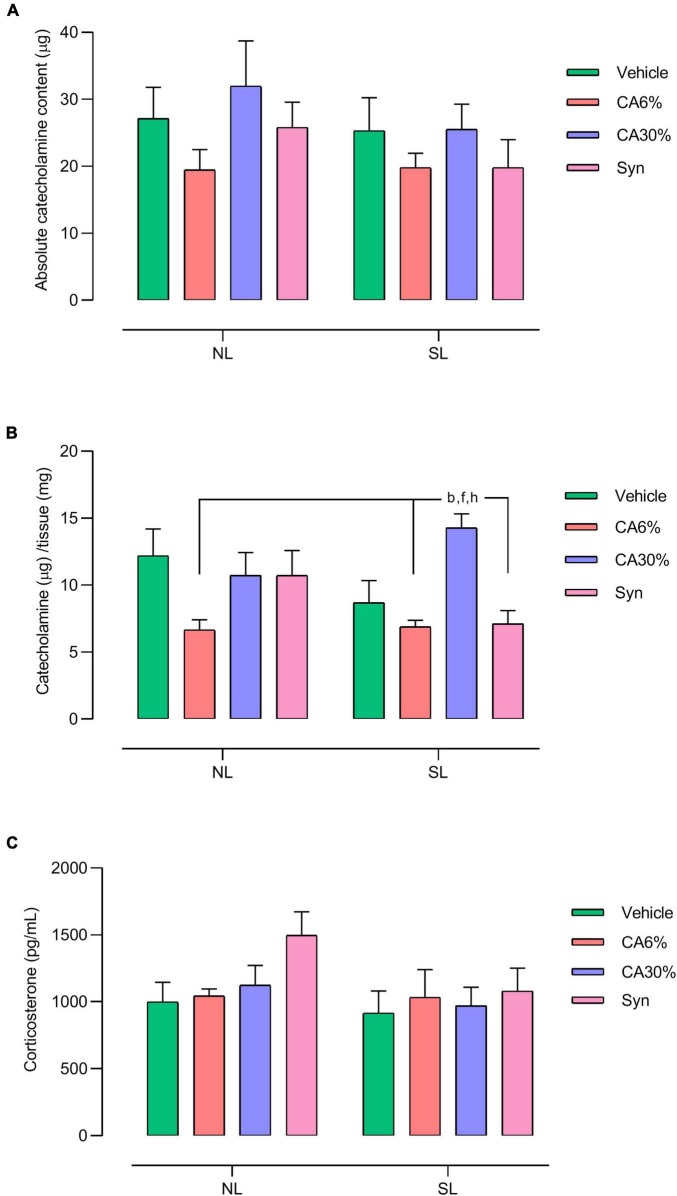
Effect of treatments with *C. aurantium* and synephrine on the medulla adrenal and plasma corticosterone. Absolute catecholamine content **(A)**, Catecholamine/tissue **(B)** and Corticosterone **(C)**. Results analyzed by bivariate analysis of variance (two-way ANOVA) followed by Tukey’s multiple comparisons test and expressed as mean ± SEM; *n* = 8–10 per group; *p* < 0.05; *b* = vs. NL-CA6%, *f* = vs. SL-CA6%, and *h* = vs. SL-Syn.

There was no significant difference in the absolute catecholamine content in the adrenal gland (Figure 6A). Regarding the relative catecholamine content, the SL-CA30% group showed an increase in the relative catecholamine content compared to the NL-CA6% (*p* ≤ 0.01), SL-CA6% (*p* ≤ 0.005), and SL-Syn groups (*p* ≤ 0.005) (Figure 6B). There was no significant difference in plasma corticosterone (Figure 6C).

### 3.8 BAT morphology

**FIGURE 7:**
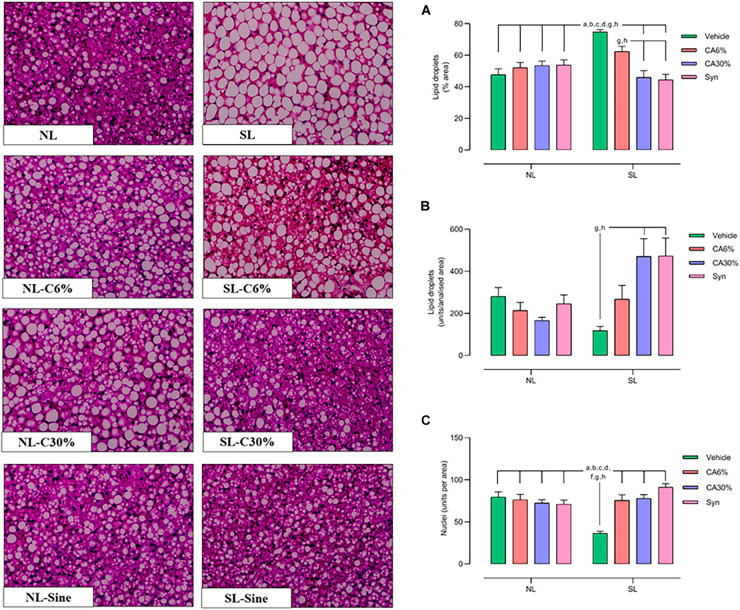
BAT histology by Hematoxylin–Eosin (HE) staining with 40× magnification. Quantitative analysis of lipid droplets and nucleus number of the BAT. Lipid Droplets (%/area) **(A)**; lipid droplets (unity/area) **(B)** and Nuclei (unity/area) **(C)**. Results analyzed by bivariate analysis of variance (two-way ANOVA) followed by Tukey’s multiple comparisons test and expressed as mean ± SEM; *n* = 6–7 per group; *p* < 0.05; *a* = vs. NL, *b* = vs. NL- CA6%, *c* = vs. NL- CA30%, *d* = vs. NL- Syn, *f* = vs. SL-CA6%, *g* = vs. SL-CA30%, and *h* = vs. SL-Syn.

In Figure 7, the **SL** group showed a higher lipid droplet sectional area (**+**56% vs. **NL**, **+**44% vs. **NL**-CA6%, **+**38% vs. **NL**-CA30% and **NL**-Syn; **+**20% vs. **SL**-CA6%; **+**63% vs. **SL**-CA30%, **+**70% vs. **SL**-Syn; ***p*** ≤ 0.0001) (Figure 7A), showed smaller lipid droplets (unit per area) (3 fold-decrease vs. **SL**-CA30% and **SL**- Syn; ***p*** ≤ 0.005) (Figure 7B) and showed fewer nuclei in the **BAT** than in the normal litter groups and in the small litter groups treated with ***C. aurantium*** and synephrine (2-fold-increase vs. **NL** and **NL**-CA6%; −98% vs. **NL**-CA30%, −94% vs. **NL**-Syn; 2-fold-increase vs. **SL**-CA6% and **SL**-CA30%; 2.5-fold-increase vs. **SL**-Syn; ***p*** ≤ 0.0001) (Figure 7C). Treatment with ***C. aurantium*** and synephrine was able to restore the lipid droplet size and quantity of nuclei in the small litter groups.

### 3.9 BAT mRNA expression

**FIGURE 8:**
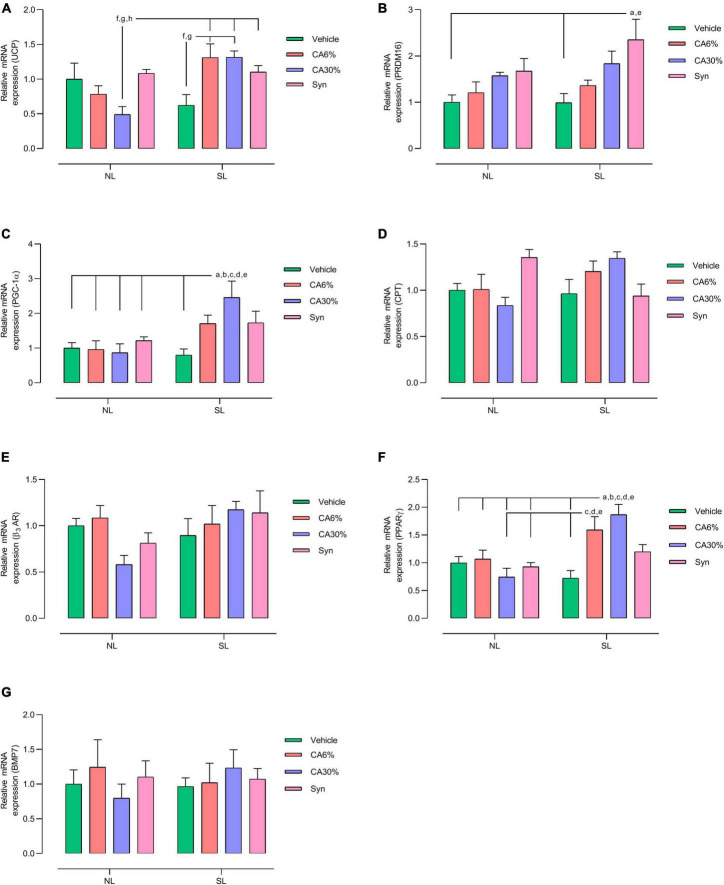
Effect of treatments with *C. aurantium* and synephrine on thermogenic factors in BAT. UCP **(A)**; PRDM16 **(B)**; PGC-1α **(C)**; CPT **(D)**; β_3_AR **(E)**; PPARγ **(F)**; BMP7 **(G)**. Mice raised in normal litter (NL); Mice raised in normal litters treated with *C. aurantium* standardized on 6% synephrine (NL-CA6%); Mice raised in normal litters treated with *C. aurantium* standardized on 30% synephrine (NL-CA30%); Mice raised in normal litters treated with synephrine (NL-Syn); Mice raised in small litter (SL); Mice raised in small litters treated with *C. aurantium* standardized on 6% synephrine (SL-CA6%); Mice raised in small litters treated with *C. aurantium* standardized on 30% synephrine (SL-CA30%); Mice raised in small litters treated with synephrine (SL-Syn). Results analyzed by bivariate analysis of variance (two-way ANOVA) followed by Tukey’s multiple comparisons test and expressed as mean ± SEM; *n* = 4–6 per group; *p* < 0.05; *a* = vs. NL, *b* = vs. NL- CA6%, *c* = vs. NL- CA30%, *d* = vs. NL- Syn, *e* = vs. SL, *f* = vs. SL-CA6%, *g* = vs. SL-CA30%, and *h* = vs. SL-Syn.

Figure 8 shows biomarkers related to thermogenesis in BAT. The NL groups did not show differences in gene expression in BAT. The SL groups treated with *C. aurantium* and synephrine showed increased gene expression of UCP-1, PRDM16, PGC-1α, and PPARγ. Treatment of the SL group with *C. aurantium*, but not synephrine, increased the mRNA expression of UCP-1 in relation to the SL group (two-fold increase SL-CA6% and SL-CA30% vs. SL; *p* ≤ 0.05 and *p* ≤ 0.05, respectively). SL groups treated with *C. aurantium* and synephrine showed higher relative mRNA expression of UCP-1 only compared to the NL-CA30% group (2.7-fold increase SL-CA6% and SL-CA30% vs. NL-CA30%; 2.2-fold increase SL-Syn vs. NL-CA30%; *p* ≤ 0.05) (Figure 8A). The SL-Syn group showed higher relative mRNA expression of PRDM-16 than the SL and NL groups (2.4-fold increase vs. SL, *p* ≤ 0.001; 2.3-fold increase vs. NL; *p* ≤ 0.01) (Figure 8B). SL-CA30% showed higher relative mRNA expression of PPARGC1α than the SL group and all the NL groups (three-fold increase vs. SL, *p* ≤ 0.005; 2.8-fold increase vs. NL, NL-CA6%, NL-CA30%, NL-Syn; *p* ≤ 0.05) (Figure 8C). The SL-CA6% group showed higher relative mRNA expression of PPAR-γ than the SL group and NL-CA30% group (2.2-fold increase vs. SL, *p* ≤ 0.01 and 2.1-fold increase vs. NL-CA30%, *p* ≤ 0.01). The SL-CA30% group showed higher relative mRNA expression of PPAR-γ than the SL group and all the NL groups (2.6-fold increase vs. SL, *p* ≤ 0.0005; 2.6-fold increase vs. NL, NL-CA6%, NL-CA30%, NL-Syn; *p* ≤ 0.05) (Figure 8F). No significant difference was observed in the gene expression of CPT (Figure 8D), ADRβ-3 (Figure 8E), or BMP7 (Figure 8G). However, treatment with *C. aurantium* (30% of synephrine) showed a close to significant increase (20%) in CPT gene expression in SL-CA30% group when compared to the NL-CA30% group (*p* ≤ 0.05) (Figure 8D).

### 3.10 BAT mitochondrial function

**FIGURE 9:**
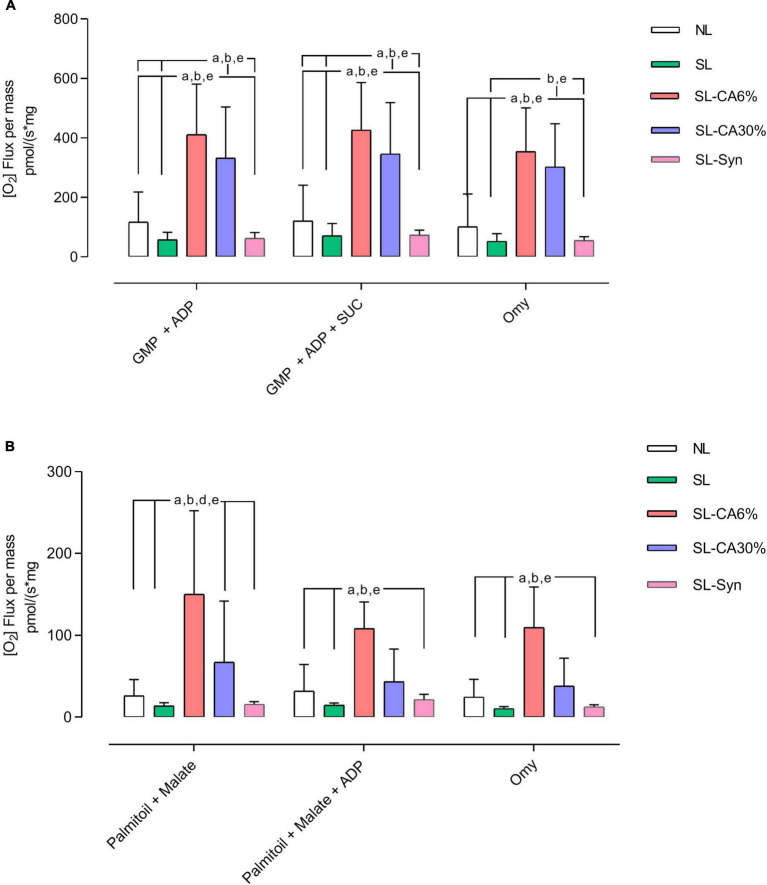
High resolution respirometry of brown adipose tissue from overfeed mice. Flux per mass with substrates pyruvate, glutamate, malate, and succinate **(A)**. Flux per mass with substrates palmitoyl-L-carnitine, malate, and ADP **(B)**. Results analyzed by One-Way ANOVA followed by Tukey’s test for multiple comparisons and expressed as mean ± SEM; *n* = 5–7 per group; *p* < 0.05; *a* = vs. NL, *b* = vs. SL, *d* = vs. SL-CA30%, and *e* = vs. SL-Syn.

Considering that SL-CA6% and SL-30% animals exhibited lower lipid droplets in BAT as well as an improvement in UCP1 gene expression, it was investigated whether mitochondrial function in this tissue could also be altered. In the presence of energy substrates, SL-CA6% promoted a significant increase in the oxidation of glutamate, malate, pyruvate, succinate and ADP, in addition to increasing the oxidation of palmitoyl-L-carnitine, malate and ADP (Figures 9A, C). The SL-CA30% group promoted a significant increase in the oxidation of glutamate, malate, pyruvate, succinate and ADP (Figure 9A). Also, SL-Syn group presented no changes in all parameters of BAT mitochondrial function evaluated.

## 4 Discussion

It is well known that overfeeding early in life causes metabolic effects in the short- and long-term, but such effects are poorly investigated in adolescence. The reduction in litter size is an effective and reproducible model of obesity (12, 15, 43). Our results demonstrated that both overweight and metabolic changes typical of obesity persist from lactation to adolescence. Moreover, the administration of *Citrus aurantium* or its active compound, synephrine, proved efficacious in ameliorating certain metabolic dysfunctions induced by postnatal overfeeding, employing distinct mechanisms.

Conceicao et al. (43) have shown that small litters (SL) become heavier when compared to normal litters (NL) on PND 30. We find at PND30, overweight and high body fat in SL group by body composition NMR analyses. Furthermore, we showed that the SL group showed higher body weight from PND4 until adolescence.

Studies were carried out upon utilization of products derived from medicinal plants and nutraceuticals for the management of obesity and metabolic disorders (44). Until this moment, no studies have demonstrated the effects of *C. aurantium* and synephrine at the same doses and period (adolescence) used in the current investigation. Here, we used a smaller dose than the one usually used in other studies because it would not be interesting to use elevate adrenergic agonist dose in adolescent animals. It is important to consider that the dose chosen was based on adult human studies, where it was demonstrated that the 9 mg of synephrine/kg body weight/day in rats is representative of the dose most used in adult humans, that is 100 mg/day of synephrine (39).

Hansen and collaborators (30) demonstrated, in adult female rats, that the administration of *C. aurantium* extract (10 or 50 mg synephrine/kg of body weight), for 28 days, did not reduce body weight. In agreement, we also observed that the SL group treated with *C. aurantium* or synephrine did not show significant differences in body weight and body fat on PND 50.

Synephrine, due to it is an adrenergic agonist effect and its similarity to ephedrine, has potential adverse effects upon the cardiovascular system (30). However, we did not show changes in heart rate or systolic or diastolic blood pressure in the treatments with *C. aurantium* extracts and synephrine.

*Citrus aurantium* has been associated with improvement in hyperglycemia (45). Although obese, the SL group had no changes in glucose homeostasis, with no significantly different in TOTG compared to the NL group. Litter size reduction programming impairs leptin signaling and causes leptin resistance at PND180 (37, 46). Corroborating these findings, the SL group showed hyperleptinemia, indicating leptin resistance in these animals as early as 50 days old. However, *C. aurantium* and synephrine slightly reduced this hyperleptinemia induced by overfeeding. In the literature, no studies were found about the effect of *C. aurantium* and synephrine on leptin secretion and signaling. Thus, precocious treatment with *C. aurantium* may prevent those animals from developing future glucose intolerance since leptin resistance can be one contributor factor to insulin resistance.

Rodrigues and collaborators (37) showed that, in PND21, the SL group had high TSH and serum thyroid hormone concentrations. However, on PND180, this group showed normal TSH and lower T3 and T4 in serum. Here, treatment with *C. aurantium* increased total T3 and free T4 compared to all NL groups in this study, and synephrine (SL-Syn) increased even more because it was higher than in the SL group. As there are no studies focusing the interplay between thyroid function and *C. aurantium* (or synephrine), more studies are needed to better understand the mechanisms involved.

As we used the same amount of synephrine in the 3 experimental groups and only the SL-CA30% group showed an increase in relative adrenal catecholamine content, it is possible that some component of *C. aurantium*, other than synephrine, can increase adrenal catecholamines, which can occur either by greater synthesis or by accumulation due to deficient secretion. We measured only tissue catecholamines, and this increase was not enough to alter cardiovascular parameters. However, this change may have resulted in a slight increase in circulating adrenaline, inducing lipolysis in white and brown adipocytes, through their interaction with β-3 adrenergic receptors (47).

Only one *in vitro* study has reported the influence of *C. aurantium* on the differentiation and activation of brown adipocytes through anti-adipogenic and thermogenic mechanisms (34). In the current study, we explored the *in vivo* anti-obesity potential of *C. aurantium* extracts and its main active component, synephrine, in brown adipose tissue dysfunction of adolescent mice programmed by early postnatal overfeeding.

The whitening of BAT occurs in obesity. In this process, BAT has increased tissue mass with large lipid droplets, low vascularization, high pro-inflammatory cytokine expression, and low UCP-1 and other thermogenesis marker expression (13, 48), causing dysfunction.

In the qualitative and quantitative analyses of BAT, we demonstrated a reduction in its mass and in the content of lipid vesicles and an increase in the number of nuclei in the SL groups treated with *C. aurantium* and synephrine, showing that the treatments normalized the BAT structure and recovered its original phenotype. We investigated some important markers of thermogenesis and activity in BAT, such as UCP-1, PRDM16, PCG-1α, CPT-1, beta-3AR, PPARγ, and BMP-7 (49). Furthermore, we found, in general, higher gene expression of UCP1, PRDM 16, PGC-1α, and PPARγ, demonstrating the thermogenic action of both *C. aurantium* and its active compound, synephrine. β-3 adrenergic receptor expression in BAT was unchanged, but we cannot ignore the effect of synephrine (β-3 adrenergic ligand) on adrenergic receptors.

PPARγ is responsible for positively regulating genes involved in lipid oxidation (CPT-1) and thermogenesis, such as UCP-1 and PGC-1α (responsible for mitochondrial biogenesis and stimulation of UCP-1 gene expression) (50). In addition, PPARγ participates in several physiological functions, such as glucose metabolism control, adipocyte differentiation regulation, and inflammatory response regulation. This receptor is considered a primary regulator of adipogenesis, controlling cell differentiation of preadipocytes into mature adipocytes (51) and acting in the direction of white and brown adipocyte differentiation (52). Here, treatment with *C. aurantium* standardized to contain 6 and 30% synephrine increased PPARγ gene expression in the SL group, but synephrine had no effect.

UCP-1 is a key marker of thermogenesis in BAT. In experimental models, the absence of UCP-1 is associated with increased body weight and decreased thermogenesis in BAT (52). In our model of obesity induced by litter size reduction, we observed BAT dysfunction and UCP-1 gene expression reduction. However, the SL-CA6% and SL-CA30% groups showed higher UCP-1 gene expression than the SL group, demonstrating that the thermogenic effect of *C. aurantium* on BAT was better than synephrine alone that only caused a higher gene expression for PRDM16, a known transcriptional co-regulator capable to directing brown adipogenesis.

Mitochondria primarily produce ATP for cellular energy by directing proton flow to ATP synthase. However, they also generate heat through a process known as “proton leak” during oxidative phosphorylation. This occurs when protons, temporarily stored in the intermembrane space, are released into the mitochondrial matrix by uncoupling proteins, reducing the membrane potential, and producing heat rather than ATP (53). Thus, the induced decrease in proton flux is mediated by an increase in UCP1 in adipose tissue, increasing heat production and potentiating weight loss. This mechanism is important as a therapeutic target in the regulation of body weight. Considering the higher expression of UCP1 in BAT, this could be one of the mechanisms of action of *C. aurantium*/synephrine in weight loss. UCP-1 activity is mainly regulated by fatty acids that represent the main energy substrates for oxidation during thermogenesis, providing NADH and FADH2 to supply the respiratory chain and ensure the proton gradient (40). Indeed, in our high-resolution respirometry analysis, we observed higher ADP-stimulated maximal respiratory rates in both oxidation protocols (A = substrates pyruvate, glutamate, malate, succinate; B = substrates palmitoyl-L-carnitine, malate, ADP) in the SL-CA6% compared to the NL and SL, which raises the possibility of a higher efficiency of the mitochondrial complexes and, consequently, improvement of cellular metabolism. On the other hand, SL-CA30% only altered the use of substrates related to carbohydrate oxidation, suggesting a less intense effect in this group compared to the effect of animals of SL-CA6% group. Isolated synephrine, in turn, did not change oxygen consumption in relation to the energy substrates studied.

In general, *C. aurantium* and synephrine increased thermogenic marker expression in BAT without modifying the cardiovascular system. The SL-CA6% and SL-CA30% group had the more effective action on BAT thermogenesis than SL-Syn due to biomarkers elevation (UCP-1 and PPAR), reduction in lipid droplets, and increase in the number of nuclei and mitochondrial function in BAT compatible to adipose tissue browning.

The modern obesogenic environment can significantly increase obesity worldwide, especially in adolescence (54). This period is a crucial stage of human development when several psychological and social changes occur in addition to the acquisition of new life habits that are the foundation for health and wellbeing in adulthood (55).

## 5 Conclusion

Our results indicate that obesity treatment in the critical period of adolescence, when the neurological system is particularly sensitive to interventions, can be a good therapeutic strategy. Even when using *C. aurantium* or synephrine at a low dose, both showed beneficial effects, reducing adipose tissue mass, and improving BAT functionality without impacting blood pressure and glucose homeostasis. With due care in extrapolating therapy from animals to humans, the set of our findings suggest that therapeutic use of these compounds can improve the metabolic profile of obese adolescents without adverse cardiovascular side effects. Further studies are necessary to better evaluate the safety of using both *C. aurantium* and synephrine.

## Data availability statement

The raw data supporting the conclusions of this article will be made available by the authors, without undue reservation.

## Ethics statement

The animal study was approved by the Animal Care and Use Committee of the Biology Institute of the State University of Rio de Janeiro. The study was conducted in accordance with the local legislation and institutional requirements.

## Author contributions

AG: Conceptualization, Formal analysis, Investigation, Writing – original draft. EM: Resources, Writing – review & editing. SS: Investigation, Writing – review & editing. BL: Formal analysis, Investigation, Writing – review & editing. IB: Investigation, Writing – review & editing. CP: Writing – review & editing, Investigation. FQ: Writing – review & editing, Formal analysis. TO: Writing – review & editing, Investigation. ÉS: Resources, Writing – review & editing. PL: Resources, Writing – original draft, Writing – review & editing. EO: Conceptualization, Funding acquisition, Project administration, Supervision, Writing – original draft, Writing – review & editing.
