# Supplementary material for: Citrus aurantium L. and synephrine improve brown adipose tissue function in adolescent mice programmed by early postnatal overfeeding
Source: Front Nutr. 2024 Jan 11;10:1278121. doi: 10.3389/fnut.2023.1278121 (PMC10809993; doi:10.3389/fnut.2023.1278121)
Supplement: Supplementary file 1 [file Data_Sheet_1.PDF]

Supplementary Material

Supplementary Figures

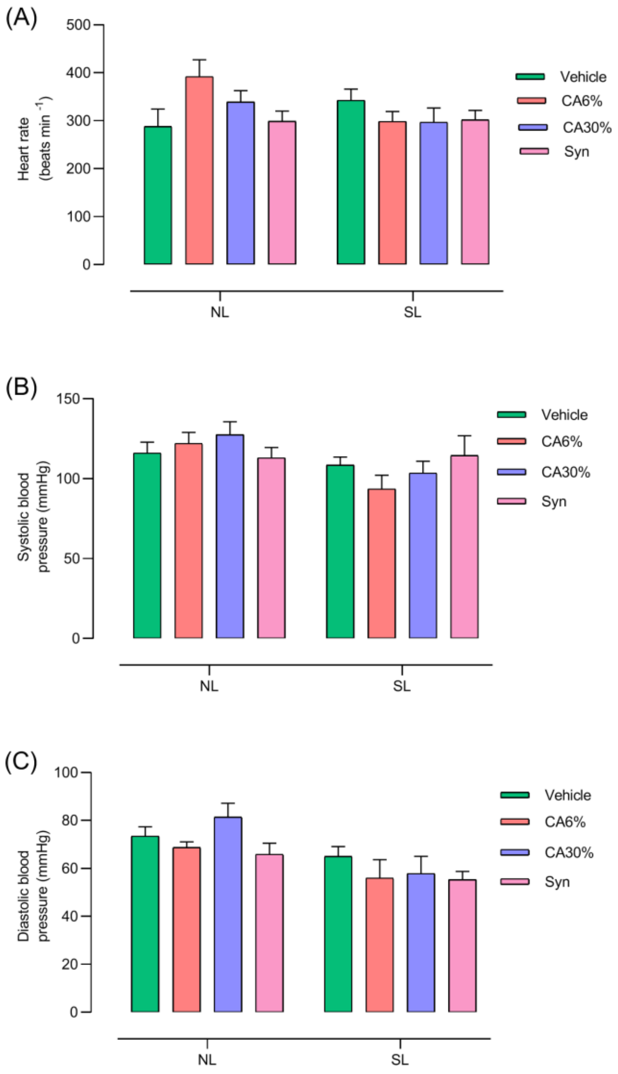

Supplementary Figure 1. Effects of treatments with *Citrus aurantium L.* and synephrine on cardiovascular parameters. The heart rate (A), systolic blood pressure (B) and diastolic blood pressure (C). Results analyzed by bivariate analysis of variance (two-way ANOVA) followed by Tukey's test for multiple comparisons and expressed as mean ± S.E.M.; *n=5-8* per group; $p<0.05$.

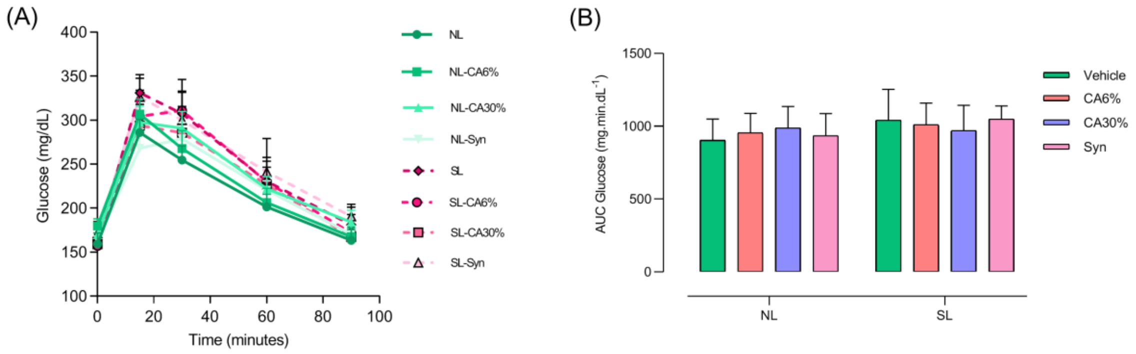

Supplementary Figure 2. Oral glucose tolerance test (OGTT) of mice raised in normal and small litters submitted to treatment with *C. aurantium* and synephrine. OGTT (A) and area under curve (AUC) of OGTT (B). Results analyzed by bivariate analysis of variance (two-way ANOVA) followed by Tukey's test for multiple comparisons and expressed as mean ± S.E.M. *n=8* per group. *p<0.05.*

Supplementary Tables

Supplementary Table 1. Treatments administered orally (gavage) with their respective doses

Treatment	Dose
Citrus aurantium L. extract standardized in 6% synephrine	25 mg/kg of weight/day
Citrus aurantium L. extract standardized in 30% synephrine	5 mg/kg of weight/day
Synephrine	1,5 mg/kg of weight/day

Abreviations: Milligram (mg); Kilogram (kg)

Supplementary Table 2. TaqMan® gene expression and reference of the sequence of specific primers used in the Real-Time PCR

**Protein**	**Gene Symbol**	**TaqMan® gene expression**	**Reference**
PPARγ	*Pparg*	Mn00440940_m1	NM_011146.3
UCP	*Ucp1*	Mm01244861_m1	NM_009463.3
CPT1	*Cpt1b*	Mm00487200_m1	NM_009948.2
PRDM	*Prdm16*	Mm00712556_m1	NM_001177995.1/NM_001291026.1
PGC-1α	*Ppargc1a*	Mm01208835_m1	NM_008904.2/NR_027710.1
β3 adrenergic	*Adrb3*	Mm02601819_g1	NM_013462.3
BMP-7	*Bmp7*	Mm00432102_m1	NM_007557.3
36B4	*Rplp0*	Mm00725448_s1	NM_007475.5

Abreviations: Peroxisome Proliferator-Activated Receptor Gamma (PPARγ); Uncoupling Protein 1 (UCP1); Carnitine palmitoyltransferase 1 (CPT1); PR-domain containing 16 (PRDM); Peroxisome Proliferator-Activated Receptor Gamma Coactivator 1-alpha (PGC-1α); Beta-3 Adrenergic Receptor (β3 adrenergic); Bone Morphogenetic Protein 7 (BMP-7); Ribosomal Protein (36b4).
